# Interlaboratory Validation of a Detection Method for Hepatitis E Virus RNA in Pig Liver

**DOI:** 10.3390/microorganisms8101460

**Published:** 2020-09-23

**Authors:** Eva Trojnar, Matthias Contzen, Dominik Moor, Anja Carl, Sabine Burkhardt, Jochen Kilwinski, Kornelia Berghof-Jäger, Sascha Mormann, Ulrich Schotte, Anne Kontek, Nadine Althof, Dietrich Mäde, Reimar Johne

**Affiliations:** 1 German Federal Institute for Risk Assessment, 10589 Berlin, Germany; Eva.Trojnar@bfr.bund.de (E.T.); Nadine.Althof@bfr.bund.de (N.A.); 2 Chemisches und Veterinäruntersuchungsamt Stuttgart, 70736 Fellbach, Germany; Matthias.Contzen@cvuas.bwl.de; 3 Bundesamt für Lebensmittelsicherheit und Veterinärwesen, 3003 Bern, Switzerland; Dominik.Moor@blv.admin.ch; 4 Bayrisches Landesamt für Gesundheit und Lebensmittelsicherheit, 91058 Erlangen, Germany; Anja.Carl@lgl.bayern.de; 5 Landeslabor Berlin-Brandenburg, 12489 Berlin, Germany; poststelle@landeslabor-bbb.de or; 6 Chemisches und Veterinäruntersuchungsamt Westfalen, 59821 Arnsberg, Germany; Jochen.Kilwinski@cvua-westfalen.de (J.K.); sascha.mormann@cvua-westfalen.de (S.M.); 7 BIOTECON Diagnostics GmbH, 14473 Potsdam, Germany; kberghof@bc-diagnostics.com; 8 Zentrales Institut des Sanitätsdienstes der Bundeswehr Kiel, 24119 Kronshagen, Germany; UlrichSchotte@bundeswehr.org; 9 Niedersächsisches Landesamt für Verbraucherschutz und Lebensmittelsicherheit, 26133 Oldenburg, Germany; anne.kontek@laves.niedersachsen.de; 10 Landesamt für Verbraucherschutz Sachsen-Anhalt, 06112 Halle (Saale), Germany; Dietrich.Maede@lav.ms.sachsen-anhalt.de

**Keywords:** Hepatitis E virus, liver, detection, validation, ring trial

## Abstract

Background: In the last years, the number of notified hepatitis E cases in humans has continuously increased in Europe. Foodborne infection with the zoonotic hepatitis E virus (HEV) genotype 3 is considered the major cause of this disease. Undercooked liver and raw sausages containing the liver of pigs and wild boar are at high risk of containing HEV. However, so far, no standardized method for the detection of HEV-RNA in pig liver is available. Methods: An international collaborative study on method reproducibility involving 11 laboratories was performed for an HEV-RNA detection method, which consists of steps of sample homogenization, RNA extraction and real-time RT-PCR detection, including a process control. Naturally contaminated pork liver samples containing two different amounts of HEV and a HEV-negative pork liver sample were tested by all laboratories using the method. Results: Valid results were retrieved from 10 laboratories. A specificity of 100% and a sensitivity of 79% were calculated for the method. False negative results were only retrieved from the sample containing very low HEV amounts near the detection limit. Conclusions: The results show that the method is highly specific, sufficiently sensitive and robust for use in different laboratories. The method can, therefore, be applied to routine food control as well as in monitoring studies.

## 1. Introduction

Infection of humans with the hepatitis E virus (HEV) genotypes 1 to 4 can result in hepatitis E, which is usually characterized as an acute self-limiting inflammation of the liver. However, fulminant fatal disease may occur in pregnant women after infection with genotype 1, and chronic infections leading to life-threatening liver cirrhosis are increasingly described in genotype 3-infected immunocompromised patients [1]. Every year about 20 million infections and more than 3.3 million clinically relevant cases are suspected due to hepatitis E worldwide [2].

HEV is a single-stranded RNA virus that is classified into the family *Hepeviridae* and the genus *Orthohepevirus* [3]. While the HEV genotypes 1 and 2 are restricted to humans only, genotypes 3 and 4 are zoonotic and widely distributed in porcine hosts [4,5]. In low income countries of Asia and Africa, where genotypes 1 and 2 are endemic, major HEV-outbreaks are mostly linked to faecally contaminated drinking water [6]. In contrast, mainly sporadic hepatitis E cases are recognized in industrialized countries in Europe and North America. Currently, an increasing incidence of confirmed hepatitis E cases is being documented in many countries of Europe [7]. A major part of these infections is considered to be a result from zoonotic virus transmission via the consumption of raw or undercooked meat, liver and meat products from infected pork or wild boar. 

Early evidence for foodborne transmission of HEV came from Japan, where human hepatitis E cases were linked to the consumption of raw deer meat [8]. Thereafter, the foodborne transmission route was confirmed by several other case reports and case/control studies [9,10,11,12]. In addition, the presence of HEV-RNA has been repeatedly confirmed in pig liver and other pork products at retail level [13,14,15,16,17,18]. A recent review compared several studies conducted worldwide, which indicated HEV detection rates between 0% and 21% for pig liver samples and between 2% and 38% for wild boar liver samples [5]. Actual surveys indicate detection rates of 5%, 12.7% and 15.5% for pig livers from Germany, The Netherlands and Spain, respectively, and up to 13.7% for wild boar livers from Italy [14,19,20,21,22]. Generally, liver samples show the highest HEV-RNA detection rates and the highest viral loads as compared to other organs and meat products without liver [5,14,19,23]. 

Several methods for HEV-RNA detection in pork products have been published so far [9,16,24,25,26,27]. However, only a few published studies on interlaboratory validation of their reproducibility and robustness under routine laboratory conditions have been available until now. As matrix-specific variations have a major impact on method performance [28], a validation using samples of the specific matrix containing defined virus amounts is crucial in order to provide reliable assays for routine analysis. In line with this, the European Food Safety Authority (EFSA) has recently ranked the development of standardized methods for detection of HEV in meat and meat products as one of the top five priorities of future foodborne virus research [29]. However, to our knowledge, only one collaborative study for validation of a method for the detection of HEV-RNA in meat products has been published so far, which focused on porcine sausages [30]. In contrast, no method for HEV-RNA detection in liver samples of pigs and wild boar has been validated for its reproducibility in routine laboratories so far, although this matrix is of high risk for HEV contamination [5,23]. 

In this work, an international collaborative study for the detection of HEV-RNA in porcine liver samples based on a previously published method [31] is presented. The method was tested by eleven independent laboratories using naturally contaminated and non-contaminated liver samples from domestic pigs. The results were analyzed, and the relative sensitivity, relative specificity and relative accuracy of the method were determined, showing its suitability for use in routine laboratories. The method can, therefore, be used in future for the screening of food liver samples for HEV, outbreak investigations as well as scientific studies.

## 2. Materials and Methods

### 2.1. Selection of Liver Samples and HEV-RNA Quantification Using RT-Droplet Digital PCR (RT-ddPCR)

Pig livers were obtained from food business operators in Germany and pre-tested by real-time RT-PCR for the presence of HEV-RNA as described [31]. Positive samples were further subjected to reverse transcription droplet digital PCR (RT-ddPCR) in order to quantify the amount of HEV RNA in the samples. The RT-ddPCR method for quantification of human norovirus RNA was used essentially as described [32]; however, it was used with HEV-specific primers and probes. Briefly, the one-step RT-ddPCR kit for probes and the QX200™ droplet digital PCR system (Bio-Rad Laboratories, Hercules, CA, USA) was used in a total reaction volume of 20 μL containing 5 μL template RNA, each 500 nM primer JHEVF (5′-GGTGGTTTCTGGGGTGAC-3′) and JHEVR (5′-AGGGGTTGGTTGGATGAA-3′) and 150 nM probe JHEVP (5′-FAM-TGATTCTCAGCCCTTCGC-BHQ1-3′) [33]. The cycling protocol consisted of reverse transcription for 60 min at 50 °C, polymerase activation for 10 min at 95 °C, followed by 80 cycles with 30 s at 94 °C, 60 s at 55 °C and 60 s at 72 °C. The ramp rate was reduced to 2 K for 1 s. The cycling protocol was finished by an enzyme inactivation step at 98 °C for 10 min and cooled down to 4 °C until analysis of the droplets in the QX200™ droplet reader. Two liver samples containing high and low amounts of HEV-RNA as well as an HEV-negative liver sample were selected and stored at −80 °C until use in the collaborative study.

### 2.2. Method for the Detection of HEV in Liver

**Figure 1:**
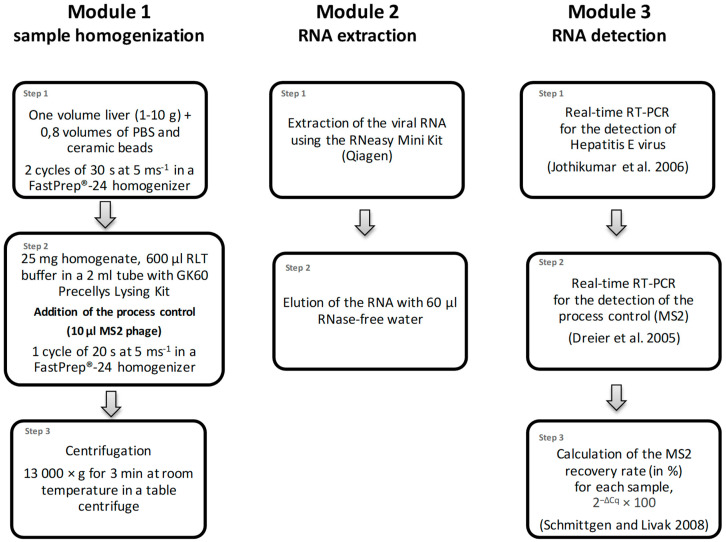
Flowchart of the method for the detection of HEV-RNA in liver. The procedure consists of three consecutive modules: (1) sample homogenization, (2) extraction of the RNA and (3) the detection of the viral HEV-RNA by real time RT-PCR.

The used method was previously published by Trojnar et al. [30] and can be divided into the three modules sample homogenization, extraction of the RNA and detection of HEV-RNA by real-time RT-PCR (Figure 1). Different amounts of starting material (1 to 10 g) were used for different applications. Generally, one part of the liver sample was homogenized in a 50 mL tube with 0.8 volumes phosphate-buffered saline (PBS, PAN Biotech GmbH, Aidenbach, Germany) and ceramic beads (1/4″ Ceramic Sphere, MP Biomedicals, Irvine, CA, USA) using a FastPrep^®^-24 homogenizer (2 × 30 s at 5 ms^−1^, MP Biomedicals). Thereafter, 25 mg of the homogenate and 10 µL of the process control (containing 5 × 10^5^ PFU of MS2 phage) were transferred to a new tube of the sample grinding GK60 Precellys Lysing Kit (Bertin Technologies SAS, Montigny-le-Bretoneux, France) and further homogenized in 600 µL buffer RLT (without addition of beta-mercaptoethanol or DTT, the buffer is part of the RNeasy Mini Kit, Qiagen, Hilden, Germany) using the FastPrep^®^-24 homogenizer (20 s at speed 5 ms^−1^). After centrifugation at 13,000× *g* for 3 min, the RNA was extracted from the entire supernatant using the RNeasy Mini Kit (Qiagen), and it was eluted with 60 µL nuclease-free water. The HEV-RNA was detected by real-time RT-PCR as described [33]. Briefly, primers JVHEV-F (250 nmol/L) and JVHEV-R (250 nmol/L) and probe JVHEV-P (50 nmol/L) were used with the Quantitect Probe RT-PCR Kit (Qiagen). For detection of bacteriophage MS2 RNA, primers MS2-TM2-F (5′-TGCTCGCGGATACCCG-3′, 250 nmol/L) and MS2-TM2-R (5′-AACTTGCGTTCTCGAGCGAT-3′, 250 nmol/L) and the probe MS2-TM2FAM (5′-FAM-ACCTCGGGTTTCCGTCTTGCTCGT-BHQ1-3′, 125 nmol/L) [34] were used with the same PCR kit. All PCRs were performed with 5 µL of extracted RNA in a total volume of 20 µL. The cycling conditions for both PCRs (performed in separate wells) were 50 °C for 30 min and 95 °C for 15 min, followed by 50 cycles of 94 °C for 10 s, 55 °C for 20 s and 72 °C for 1 min. The MS2 recovery rate (in %) was calculated by comparing the bacteriophage MS2-specific RT-PCR *Cq* value of a sample with that derived from RNA directly isolated from 10 µL of the original bacteriophage preparation with the RNeasy Mini Kit (Qiagen), using the formula 2^−ΔCq^ × 100 [35]. The recovery rate for a 1:10 diluted sample was calculated by comparison with the *Cq* value of a 1:10 diluted RNA preparation of the original bacteriophage preparation using the same formula. Samples showing a recovery rate > 1% were considered as valid as suggested by standardized methods for food-borne virus detection [36].

### 2.3. Collaborative Study for Method Validation

For the ring trial, two pig liver samples containing different amounts of HEV-RNA and one HEV-negative pig liver sample were homogenized in bulk and then aliquoted into individual samples of 500–1000 mg liver homogenate, which were stored at −80 °C. Some of the samples were thawed and pre-tested before the ring trail started. Ten independent laboratories from Germany and one laboratory from Switzerland participated in the validation study. Each laboratory received a set of twelve blinded samples of liver homogenates, which represented 4 samples of contamination level D_0_ (HEV-RNA-negative), D_1_ (lowly contaminated) and D_2_ (highly contaminated) (Table 1). In addition, an HEV-positive control, a bacteriophage MS2 process control (200 µL suspension of 5 × 10^7^ PFU/mL) and the main reagents needed for the procedure were provided to all laboratories. Samples were shipped overnight on dry ice and were stored at −18 °C until sample processing, which should be performed within a few days. The laboratories were given 6 weeks for the entire processing. Since the samples arrived pre-homogenized, the analysis protocol was started with Step 2 of the homogenization procedure using a sub-sample of 25 mg from each sample (Figure 1). In order to monitor the method performances, several controls were included as described by Althof et al. [30]. Each sample was analyzed in a single reaction, undiluted and in 1:10 dilution. Participating laboratories had to report all *Cq* values of the HEV-specific PCR for the undiluted and diluted (1:10) samples, as well as the MS2 recovery rates. If the recovery rate of the undiluted sample was below 1%, the result of the 1:10 dilution was used. Samples showing typical exponential amplification curves and *Cq* values < 50 in the HEV-specific PCR were considered positive. HEV-negative sample results with MS2 recovery rates of ≥1% were considered as valid.

### 2.4. Data Analysis

In order to evaluate the method performance, all reported results of each laboratory were screened in a first step for plausibility and general laboratory performance, and results of obviously ineligible laboratories were excluded. The relative sensitivity (in %) was calculated from the data by: (number of samples assessed as HEV-positive [D_1_ + D_2_]/total number of all evaluable HEV-contaminated samples [D_1_ + D_2_])*100. The relative specificity (in %) was calculated as: (number of samples assessed as HEV-negative/total number of all evaluable not contaminated samples)*100. The relative accuracy (in %) was calculated as: (number of samples assessed correctly [D_0_ + D_1_ + D_2_]/total number of samples [D_0_ + D_1_ + D_2_]) * 100.

## 3. Results

### 3.1. Pre-Testing of the Samples

Three pig liver samples were selected and analyzed by RT-ddPCR for the quantification of the amount of HEV-RNA. While one sample (contamination level [D_0_]) was HEV-RNA-negative, the highly contaminated sample (contamination level [D_1_]) contained an average number of 2.5 × 10^6^ genome equivalents (GE)/g and the lowly contaminated sample (contamination level [D_2_]) contained an average number of 3.2 × 10^3^ GE/g. The detailed results of all samples are presented in Table 1.

**Figure 2:**
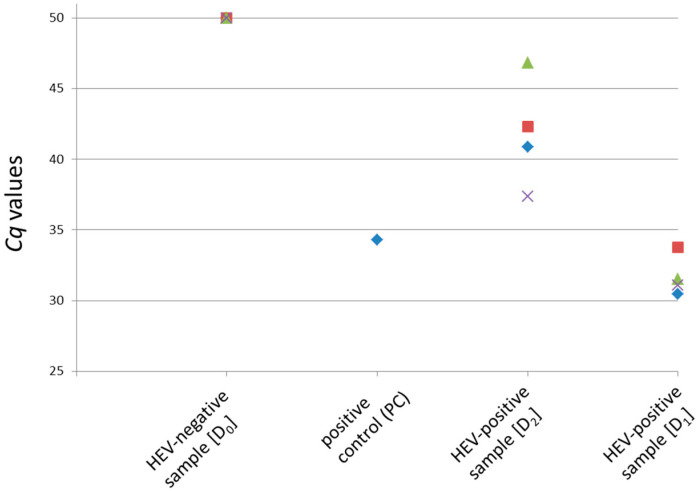
Pre-testing of the original material used in the ring trial using the described method for detection of HEV-RNA in pig liver. One positive control (PC, previously HEV positive-tested RNA of a liver sample) and four aliquots each of the contamination levels D_0_ to D_2_ were analyzed. The *Cq* values derived by the HEV-specific real-time RT-PCR are shown. A *Cq* value of 50 indicates a negative result.

Immediately prior to the ring trial, aliquots of the stored liver homogenates were tested again by exactly following the described protocol for detection of HEV-RNA in pig liver. Four aliquots of each contamination level (D_0_, D_1_ and D_2_) were analyzed, along with one positive control. Figure 2 summarizes the results of the pre-testing. The positive control showed a *Cq* value of 34.3, whereas all four samples of the contamination level D_0_ were negative. Contamination level D_1_ resulted in *Cq* values between 30.5–33.8. The *Cq* values of contamination level D_2_ ranged between 37.4 and 46.8, indicating an HEV-RNA amount near the detection limit of the method.

### 3.2. Collaborative Study

Eleven independent laboratories were included in the collaborative study. Each of the laboratories received twelve blinded liver homogenate samples, which consisted of four samples for each of the three contamination levels D_0_, D_1_ and D_2_ (Table 1). Additional material was sent to the laboratories as specified in the methods section. All laboratories used the protocol and devices as specified in the methods section beginning at step 2 of the homogenization procedure (Figure 1) and RNA extraction. Furthermore, the real-time RT-PCR was performed as described in the methods section; however, eight different PCR machines were used as listed in Supplementary Table S1. All data were returned in due time.

**Figure 3:**
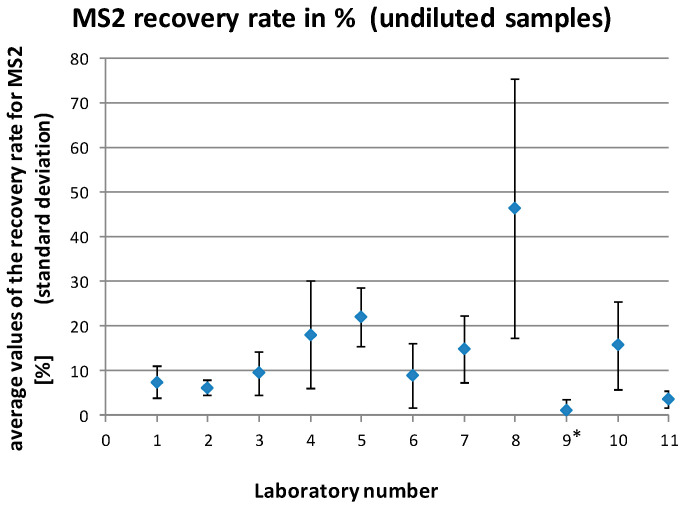
Average MS2 recovery rates (with standard deviations) for the individual laboratories for all analyzed undiluted samples. * laboratory no. 9 was excluded from the final data analysis.

**Figure 4:**
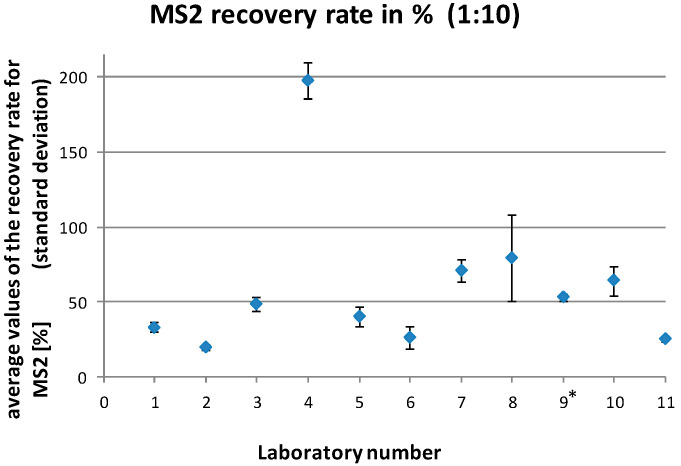
Average MS2 recovery rates (with standard deviations) for the individual laboratories for all analyzed diluted (1:10) samples. * laboratory no. 9 was excluded from the final data analysis.

The MS2 recovery rates reported by the laboratories ranged between 0% and 90.8% for the undiluted samples and 0% and 303.1% for the diluted samples (1:10). This resulted in mean recovery rates for the individual laboratories of 1.0%–46.3% for the undiluted samples and 26.2%–197.1% for the diluted samples (1:10), respectively (Figure 3 and Figure 4).

**Figure 5:**
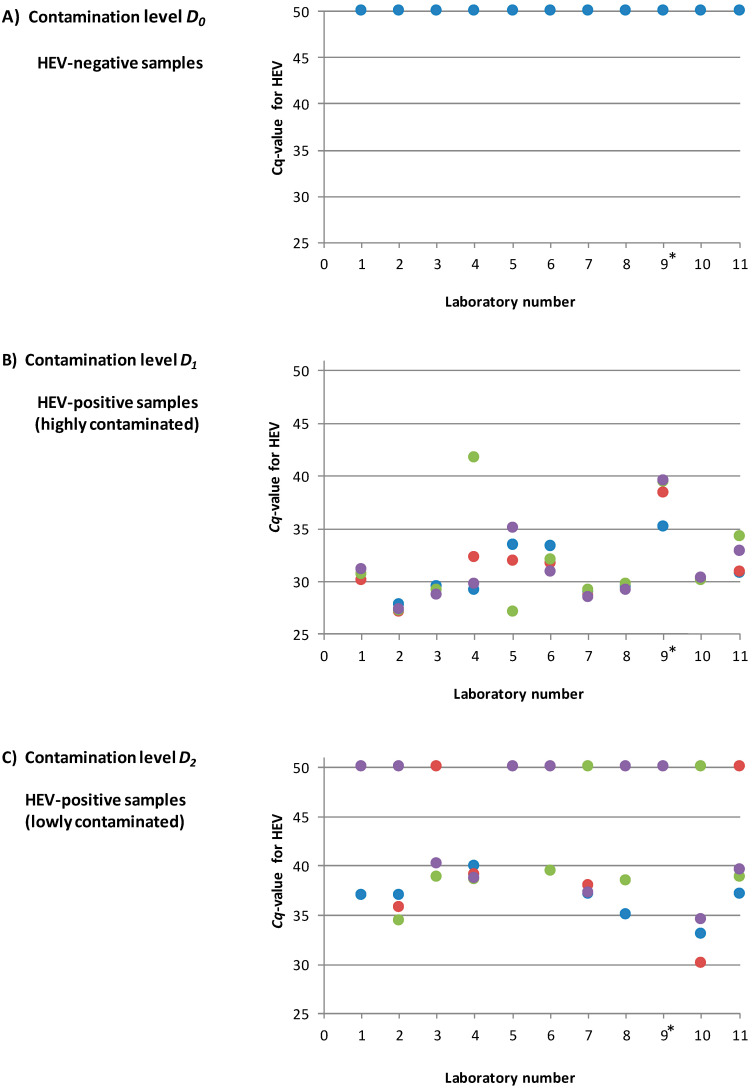
Overview of the reported results for the three different contamination levels D_0_, D_1_ and D_2_ for all participating laboratories (four samples for each contamination level per laboratory). (**A**): Contamination level D_0_ (HEV-negative). (**B**): Contamination level D_1_ (high HEV contamination) (**C**): Contamination level D_2_ (low HEV contamination near the detection limit). The *Cq* values derived by the HEV-specific real-time RT-PCR are shown. A *Cq* value of 50 indicates a negative result. * laboratory no. 9 was excluded from the final data analysis.

An overview of the reported results for HEV is shown in Figure 5. The individual *Cq* values for HEV for all laboratories are listed in Supplementary Table S2. All non-contaminated samples (D_0_) were correctly identified as HEV-negative by all participants. The highly HEV-contaminated samples (D_1_) were all correctly identified as HEV-positive with *Cq* values between 27.1 and 41.7. For the samples with the low contamination level near the detection limit (D_2_), 52% (23/44) were identified as positive with *Cq* values between 34.4 and 40.2. Thus 48% (21/44) of this contamination level were scored false-negative.

### 3.3. Data Analysis and Calculation of Method Performance Parameters

Data analysis indicated systematic problems in one of the laboratories (no. 9). This laboratory showed inacceptable MS2 recovery rates (<1%) for many of the samples and clear different *Cq* values for the positive control as compared to the other laboratories. An investigation turned out that an in-house positive control (instead of the control submitted with the samples) was used by this laboratory, which led to a limited comparability with the values of the other laboratories. For this reason, and because the recovery rates also indicated a general problem of the laboratory with handling of the method, the results of this laboratory were not included in the final data analysis.

The remaining ten laboratories provided valid results, which were all incorporated into the data analysis (120 samples = 100%). These data were used to determine the method performance parameters. The relative specificity of the method was calculated to be 100%, the relative sensitivity 79% and the accuracy 86%.

## 4. Discussion

The number of HEV infections has increased in several European countries during the last few years [7]. As most of these infections are considered to be acquired locally, most likely by consumption of meat products from infected pigs and wild boar, appropriate HEV detection methods for food control are urgently needed. Because reservoir animals do not show any symptoms of acute illness after HEV infection [37,38], laboratory methods have to be used for screening of animals or food for this virus. Reliable methods should be suitable for use in routine laboratories, which requires their validation in collaborative studies. So far, only one collaborative study for the validation of a method for HEV-RNA detection in meat products has been published [30]. However, this method focuses on analysis of pork sausages and is very laborious and time-consuming. In addition, it uses hazardous substances (TRI Reagent^®^ Solution), which require handling under specific safety conditions. Therefore, our study aimed at the interlaboratory validation of an easy-to-perform method for HEV-RNA detection in pig liver.

Several methods for detecting HEV-RNA in pig liver, meat and pork products have been published [9,16,24,25,26,27]. We selected a previously published method [31], which is easy to perform and quick, and based on widely available commercial reagents and devices. During the ring trial, the results of only one laboratory indicated problems with the handling of the method, whereas the remaining ten laboratories obtained exclusively valid results. The method uses a homogenization step directly at the beginning, which enables HEV identification even in the case of uneven distribution of HEV in the sample. As determined in our study, the method was able to detect as low as 3.2 × 10^3^ GE/g liver homogenate in 52% of the samples, indicating a high sensitivity. As a general limitation, the method cannot distinguish if infectious or already inactivated HEV is present in a sample. The development of broadly applicable techniques for infectivity determination has to be awaited in the future in order to solve this problem [39].

Several controls have been implemented in the method in order to screen its performance for each investigated sample. One of the most important controls is the process control, which monitors the efficiency of the whole analytic process by addition of a heterologous virus to each sample. Here we used the bacteriophage MS2, which is a small, non-enveloped, single-stranded RNA virus similar to HEV and has already been used as a PCR and extraction control for other foodborne viruses [16,34,40]. Due to the relatively simple cultivation method for this bacteriophage and its usability in laboratories with no restrictions at low biosafety levels, it can easily be propagated in many laboratories. While in some more complex matrices, such as sausages, the use of the bacteriophage MS2 as a process control has been shown to be somewhat problematic [15,16,30]; its usability in liver homogenates seems to be possible as determined in our study.

The validation study showed that the method is robust and has a good method performance. Most importantly, the specificity of 100% excludes the appearance of false-positive results. The relative sensitivity of 79% may indicate that the method should be improved in the future in order to exclude false-negative results. However, a closer view at the data shows that the false-negative results have been exclusively retrieved by analysis of samples of contamination level D_2_, which contained only very low HEV amounts representing only seven genome copies per PCR tube. A percentage of negative results is, therefore, to be expected for these samples by statistical reasons. The results show that the contamination levels used were chosen properly to assess the performance characteristics of the method as suggested by international standard protocols [41].

It should be mentioned that our interlaboratory validation also has some limitations. In order to ensure homogeneous material to be tested by all laboratories, the first step of liver preparation, which may introduce further variation of results, had to be excluded. As reagents and controls were prepared by one partner and distributed to all participating laboratories, possible variations arising from the differences in their preparation have also not been assessed here. In addition, only three samples have been analyzed in the collaborative study, which is in accordance with suggestions from international standard protocols [41], but higher variability of results may occur by the inclusion of other samples containing other HEV strains or slightly different matrix compositions.

Based on the high degree of usability and reproducibility as determined in this study, the method described here appears to be applicable for routine detection of HEV-RNA in naturally contaminated pig liver. It can, therefore, be applied in routine screening for HEV in liver from pork and wild boar as well as in scientific studies and outbreak investigations.

## Figures and Tables

**Table 1:** Overview of the sample set used in the validation study.

Contamination Level	GE/5 µL RNA (as Used in PCR)	GE/g Liver Homogenate	Number of Samples/Lab	Total Number of Samples/Study
[D_0_]	0	0	4	44
[D_1_] highly contaminated	5860	2.5 × 10^6^	4	44
[D_2_] lowly contaminated	7.4	3.2 × 10^3^	4	44
			∑: 12	∑: 132
Positive control	900	3.9 × 10^5^	1	11

GE: genome equivalent.

## Supplementary Materials

The following are available online at https://www.mdpi.com/2076-2607/8/10/1460/s1, Table S1: PCR machines used by the laboratories for detection of HEV and MS2 by real-time RT-PCR, Table S2: HEV-specific *Cq* values of all participating laboratories for the detection of hepatitis E virus RNA.
