# Supplementary material for: Interlaboratory Validation of a Detection Method for Hepatitis E Virus RNA in Pig Liver
Source: Microorganisms. 2020 Sep 23;8(10):1460. doi: 10.3390/microorganisms8101460 (PMC7598171; doi:10.3390/microorganisms8101460)
Supplement: Supplementary file 1 [file microorganisms-08-01460-s001.pdf]

Supplementary Material
S1-S2

Interlaboratory Validation of a Detection Method for Hepatitis E Virus RNA in Pig Liver

Trojnar *et al.* (2020), *Microorganisms*

Table S1: PCR machines used by the laboratories for detection of HEV and MS2 a by real-time RT-PCR.

Machine model	**manufacturer**	Number of users
ABI 7500	**Applied Biosystems**	2
RotorGene RG-6000	**Corbett Research**	1
LightCycler 480	**Roche Diagnostics**	1
LightCycler 96	**Roche Diagnostics**	1
MX3005P	**Stratagene**	1
RotorGene Q	**Qiagen**	1
BioRad CFX 96	**Bio Rad Laboratories**	3
Agilent AriaMX	**Agilent**	1

Table S2: HEV-specific *Cq* values of all participating laboratories for the detection of hepatitis E virus RNA.

Sample ID	dilution	HEV contamination	Laboratory 1	Laboratory 2	Laboratory 3	Laboratory 4	Laboratory 5	Laboratory 6	Laboratory 7	Laboratory 8	Laboratory 9*	Laboratory 10	Laboratory 11
**1**	1:1	-	-	-	-	-	-	-	-	-	-	-	-
**1**	1:10	-	-	-	-	-	-	-	-	-	-	-	-
**2**	1:1	+	37	36,93	-	39,9	-	-	37,15	35	-	33,07	37,05
**2**	1:10	+	-	-	-	-	-	41,48	-	-	-	31,71	39,64
**3**	1:1	+++	31,12	27,87	29,53	29,2	33,4	33,34	29,1	29,57	35,17	30,36	30,85
**3**	1:10	+++	35,75	31,63	32,44	31,1	40,55	31,47	31,31	32,44	39,37	31,66	32,56
**4**	1:1	+++	30,06	27,08	29,15	32,3	31,98	31,75	28,66	29,18	38,36	30,07	30,87
**4**	1:10	+++	31,52	31,04	32,4	34,5	35,02	35,25	30,84	31,91	-	31,12	33,2
**5**	1:1	-	-	-	-	-	-	-	-	-	-	-	-
**5**	1:10	-	-	-	-	-	-	-	-	-	-	-	-
**6**	1:1	+	-	35,72	-	39	-	-	37,97	-	-	30,05	-
**6**	1:10	+	-	39,84	-	-	-	-	-	-	-	31,91	-
**7**	1:1	-	-	-	-	-	-	-	-	-	-	-	-
**7**	1:10	-	-	-	-	-	-	-	-	-	-	-	-
**8**	1:1	+	-	34,43	38,8	38,6	-	39,43	-	38,46	-	-	38,82
**8**	1:10	+	-	37,89	-	39,1	-	-	-	-	-	-	-
**9**	1:1	+++	30,68	27,27	29,16	41,7	27,14	32,08	29,15	29,77	39,41	30,14	34,25
**9**	1:10	+++	33,05	30,48	32,4	32,9	29,96	34,4	30,74	32,03	-	31,17	35,07
**10**	1:1	-	-	-	-	-	-	-	-	-	-	-	-
**10**	1:10	-	-	-	-	-	-	-	-	-	-	-	-
**11**	1:1	+	-	-	40,17	38,7	-	-	37,23	-	-	34,49	39,58
**11**	1:10	+	-	-	-	-	-	-	-	-	-	-	-
**12**	1:1	+++	31,17	27,31	28,78	29,8	35,07	30,94	28,45	29,22	39,56	30,36	32,91
**12**	1:10	+++	33,75	30,71	30,82	32,2	37,76	31,67	30,56	32,29	-	31,26	33,83
**Positive HEV RT-PCR control**	1:1	++	33,73	29,57	31,25	39,3	32,85	33,48	31,37	33,58	36,6	32,82	35,73
	1:10	++	34,38	n.d.	32,97	35,7	35,17	34,77	33,12	34,38	35,32	32,82	35,94
**Positive MS2 RT-PCR control**	1:1	-	-	-	-	-	-	-	-	-	-	-	-
	1:10	-	-	-	-	-	-	-	-	-	-	-	-
**Negative RT-PCR control**		-	-	-	-	-	-	-	-	-	-	-	-
**Negative process control**		-	-	-	-	-	-	-	-	-	-	-	-

- HEV-negative sample, + HEV-positive sample at the LOD, +++ HEV-positive sample above the LOD
*laboratory no. 9 was excluded from the final data analysis.
